# Food Environment and Its Effects on Human Nutrition and Health

**DOI:** 10.3390/nu16111733

**Published:** 2024-06-01

**Authors:** Alicia del Carmen Mondragon Portocarrero, Jose Manuel Miranda Lopez

**Affiliations:** Laboratorio de Higiene, Inspección y Control de Alimentos, Departamento de Química Analítica, Nutrición y Bromatología, Campus Terra, Universidade da Santiago de Compostela, 27002 Lugo, Spain; alicia.mondragon@usc.es

## 1. Introduction

The concept of a healthy diet is not a static definition; over the years, it has been molded to scientific knowledge. Recently, international organizations have updated their concept of a healthy diet, defining it as one that promotes all dimensions of health and individual well-being; has low environmental pressure and impact; is accessible, affordable, safe, and equitable; and is also culturally acceptable [1], as consumers are generally reluctant to change their eating habits [2]. Therefore, the ways in which food is produced, processed, packaged, distributed, labeled, priced, consumed, or wasted represent areas that, while not included in the traditional concept of human nutrition, affect human health, and need to be considered [3]. Thus, an ideal diet should aim to achieve optimal growth and development for all individuals and promote physical, mental, and social well-being at different stages of life, reducing the risk of diet-related noncommunicable diseases and supporting the preservation of biodiversity and environmental health [4].

Therefore, in addition to the proportions of foods that can be included in the human diet, there are other conditioning factors that can affect human health [5]. Thus, the dietary contributions of bacteria [6], viruses [7], fungi [8,9], food additives [10], chemical substances [11,12], or exosomes can exert important effects on human health [13]. Similarly, lifestyle factors, such as meal timing, cuisine, and type of work, also have an important effect on human health. It should not be forgotten that through nutrition, we can alleviate or cure diseases that affect organs far away from the digestive system, as in the case of nonfood allergies [14], respiratory viral infections [7], heart diseases [15], diseases of the nervous system, and neurological disorders [16,17].

In view of this, the objective of this Special Issue was to provide an update of knowledge on those environmental factors that play an essential role in human nutrition and, therefore, in the health of individuals, especially about metabolic diseases. A total of nine research articles and five reviews covering numerous topics of great interest and topicality in human nutrition and dietetics were included. Articles investigating the influence of food outlets, the workplace of schedules, and the intake of traditional Asian foods or functional foods, including aspects related to epigenetics or metagenomics, were included. In addition, the information published included different stages of life, such as infant nutrition, the prevention of metabolic diseases in adults, and the prevention and monitoring of malnutrition in the elderly population.

## 2. An Overview of the Published Articles

Zhou et al. (Contribution 1) present an interesting paper investigating malnutrition rates and their relationship to the length and quality of hospital stay in a total of 5821 hospitalized adult patients. The effect of a nutrition intervention program carried out by volunteers in China called *Nutrition Day* was evaluated in these patients. A malnutrition rate of 22.8% was found, and the results showed that after the implementation of *nutrition day*, the diet and nutritional status of hospitalized patients improved, which had important effects on the length and quality of hospital stay.

In another article, Zhang et al. (Contribution 2) assessed the home eating environment of elderly people, analyzed its association with their body mass index (BMI), and provided recommendations for improving the home eating environment for a total of 1764 elderly people. The results showed that senior people living alone had a greater BMI than those who did not live alone, and highlighted the impact of the home environment, living conditions, and availability of canned food on the BMI of senior people.

Regarding the influence of food sales channels on human nutrition, two interesting papers have been included in this Special Issue. On the one hand, Valenčič et al. (Contribution 3) developed and tested a mobile application (called *SnackTrack*) to investigate the association between physical and digital environments on snack choice, which was tested by 188 users. The results showed that the time at which the snack was obtained did not have a relevant effect on the health of the snacks chosen. In contrast, unhealthy background images seemed to encourage the choice of healthier snacks, thus showing that environmental cues have a strong influence on consumers’ food choices. In another article, Needham et al. (Contribution 4) developed a new comprehensive method for ranking food retail environments. This method was tested with the aim of assessing its influence on obesity in a total of 47,245 users. The results obtained showed that increasing access and availability to a diverse range of food outlets, particularly healthy foods, improves consumer health.

Permatasari et al. (Contribution 5) investigated the relationship between the consumption of a typical Asian food, edible bird’s nest (EBN), and the development of metabolic diseases. This study involved three approaches: in silico, in vitro, and in vivo molecular docking simulations in rats fed with cholesterol- and fat-rich diets. Interestingly, in vivo studies revealed significant improvements in the lipid profile, blood glucose levels, enzyme levels, and inflammatory biomarker levels in rats given a high-dose dietary supplement of EBN. Additionally, dietary supplementation with high-dose EBN increased peroxisome-proliferator-activated receptor-gamma coactivator and reduced β-hydroxy β-methylglutaryl-CoA reductase. Based on these results, EBN may be a potential functional food for people with metabolic syndrome (MS).

Zand et al. (Contribution 6) investigated the effect of the long-term exposure (30 and 90 days) to a commonly used artificial food coloring agent (tartrazine) in mice. The applied dose of tartrazine was termed the human equivalent dose for the acceptable daily intake (ADI). After this intervention, the impact of tartrazine intake on the transcription of epigenetic effectors, members of the DNA methyltransferase and histone deacetylase families, was evaluated. The results revealed a significant upregulation of genes in the analyzed organs in various patterns following tartrazine intake at the ADI.

Park and Liu (Contribution 7) examined the possible causal relationship between noodle consumption and the risk of MS in adult populations from urban (58,701) and rural (13,598) hospitals. In subjects with high noodle consumption, a higher caloric intake was observed, with lower carbohydrate and higher fat, protein, and sodium intakes and lower calcium, vitamin D, vitamin C, and flavonoid intakes. Together, these proportions indicate lower diet quality. The glycemic index and glycemic load of daily meals were much greater in the high noodle intake group than in the low noodle intake group. In conclusion, noodle intake had a positive causal association with MS in Asian adults.

Nurkolis et al. (Contribution 8) investigated the potential effects of an aqueous extract of *Caulerpa racemosa* (AEC) on markers of cardiometabolic syndrome and modulation of the gut microbiome in mice fed a cholesterol- and fat-enriched diet. The administration of high doses of AECs improved blood lipid and glucose profiles and reduced the levels of several markers of endothelial dysfunction. In addition, a correlation between specific gut microbiomes and biomarkers associated with cardiometabolic diseases was obtained. In vitro assays revealed the antioxidant properties of AECs, while in vivo assay found that AEC intake contributed to the management of MS through the regulation of oxidative stress, inflammation, and endothelial function and the modulation of the gut microbiota (GM).

To investigate the association between the GM and body weight, Sinisterra-Loaiza et al. (Contribution 9) evaluated the dietary intake of 108 individuals with different weight statuses and analyzed their GM profiles to determine their GM composition and functionality, as well as their associations with BMI and diet. Correlation analysis revealed that adequate nutritional recommendations for fiber were associated with increased abundances of *Prevotella copri*, *Faecalibacterium prausnitzii*, *Bacteroides caccae*, and *Roseburia faecis*, which, together, can be considered an improvement in the GM profile. Benefits were also found at the GM level when subjects ingested amounts of monosaturated fatty acids within current recommendations.

This Special Issue also included a total of five review articles which, like the experimental articles, covered a wide variety of topics. Martino et al. (Contribution 10) reviewed nutrient-driven epigenetic alterations induced by miRNAs derived from food absorbed into the circulatory system that potentially contribute to the modulation of health and disease. The results showed that the absorption of foods carrying exogenous miRNAs modifies redox homeostasis and inflammatory conditions underlying pathological processes, such as type 2 diabetes mellitus (T2D), insulin resistance, MS, and cancer.

Padhani et al. (Contribution 11) reviewed five studies covering 3387 participants that evaluated the effectiveness of specially formulated foods (SFFs) compared to non-food-based approaches for managing malnutrition in children >6 months old. The main conclusion of the review is that SFFs may be a useful tool that can be beneficial for children with moderate wasting, which occurs on a frequent basis in humanitarian contexts.

López-Santamarina et al. (Contribution 12) reviewed the effects of the work environment on the composition and functionality of workers’ GM, focusing especially on unconventional work schedules and environments. They also analyzed whether probiotic supplements, through modulation of the GM, can moderate the effects of sleep disturbances on the immune system, as well as restore the dysbiosis caused by unusual work schedules. The results showed that rotating shift work is associated with an increased risk of various metabolic diseases, such as obesity, MS, and T2D. In addition, sleep disturbances induce both physiological and psychological stress responses and alter the healthy functioning of the GM, thus triggering an inflammatory state.

Nelson and Stice (Contribution 13) reviewed neural vulnerability factors that increase the risk of unhealthy weight gain. In conclusion, their review found potential to create novel, comprehensive, multicomponent intervention approaches that address all components of the contextualized neural vulnerability model of obesity. Understanding unique risk pathways may also create opportunities to personalize prevention efforts by identifying combinations of risk factors and tailoring intervention components to the unique needs of the individual.

Finally, Ezzatpour et al. (Contribution 14) reviewed the potential effect and influence of the gut virome, which interacts with the bacterial microbiota. The effectiveness, applicability, and potential of virome-rectification-based therapies for different diseases, including metabolic diseases, were also investigated. As the main conclusion of this review, stool metagenomic investigations should include the identification of bacteria and phages, as well as their correlation networks, to better understand gut microbiota activity in metabolic disease progression.
